# Linkage analysis merging replicate phenotypes: an application to three quantitative phenotypes in two African samples

**DOI:** 10.1186/1753-6561-5-S9-S81

**Published:** 2011-11-29

**Authors:** Anthony L Hinrichs, Robert C Culverhouse, Brian K Suarez

**Affiliations:** 1 Department of Psychiatry, Washington University School of Medicine, 660 South Euclid Ave., Campus Box 8134, St. Louis, MO 63110, USA; 2 Department of Medicine, Washington University School of Medicine, 660 South Euclid Ave., St. Louis, MO 63110, USA; 3 Division of Biostatistics, Washington University School of Medicine, 660 South Euclid Ave., St. Louis, MO 63110, USA; 4 Department of Genetics, Washington University School of Medicine, 660 South Euclid Ave., St. Louis, MO 63110, USA

## Abstract

We report two approaches for linkage analysis of data consisting of replicate phenotypes. The first approach is specifically designed for the unusual (in human data) replicate structure of the Genetic Analysis Workshop 17 pedigree data. The second approach consists of a standard linkage analysis that, although not specifically tailored to data consisting of replicate genotypes, was envisioned as providing a sounding board against which our novel approach could be assessed. Both approaches are applied to the analysis of three quantitative phenotypes (Q1, Q2, and Q4) in two sets of African families. All analyses were carried out blind to the generating model (i.e., the “answers”). Using both methods, we found numerous significant linkage signals for Q1, although population colocalization was absent for most of these signals. The linkage analysis of Q2 and Q4 failed to reveal any strong linkage signals.

## Background

The simulated mini-exome data for Genetic Analysis Workshop 17 were derived from the pilot3 study of the 1000 Genomes Project (http://www.1000genomes.org). The data consist of information on 3,205 genes containing 24,487 single-nucleotide polymorphisms (SNPs) on 697 unrelated individuals drawn from 7 population samples. The family data consist of 8 extended pedigrees (varying in size from 73 to 128 individuals), also with a total of 697 individuals. The pedigrees were seeded with 202 founders, who were described as having been drawn at random from the 697 unrelated individuals. Two hundred replicates were provided for both the unrelated individuals data set and the family data set. A noteworthy feature of these data is that the SNP genotypes from replicate to replicate are identical. In other words, for any given individual over all replicates, each person is represented as though he or she is one of 200 monozygotic twins.

Because of the unusual nature of the data sets, we elected to explore an innovative approach that takes into account the data’s replicate structure. As a complement to this approach, we also carried out an old-fashioned linkage analysis that does not take into account the fact that the families are not genetically independent from one replicate to another.

Because our analyses were blind to the generating model, we began our investigation by obtaining some descriptive statistics. We started with the genotypes of the *N* = 697 unrelated individuals and quickly discovered that a large proportion (38.5%) of the 24,487 SNPs have a frequency of 1/(2*N*); that is, only one copy of the variant exists in the entire set of unrelated individuals, and fully 87.2% have a minor allele frequency less than 5.0%. Under usual circumstances, this collection of SNPs would have such a low polymorphic information content as to render ordinary linkage analysis powerless to identify genes involved in the quantitative phenotypes of interest. Fortunately for the family data set, the data simulators provided fully informative identity-by-descent (IBD) matrices for each pair of individuals at each gene. These IBD scores were given as 0, 0.5, and 1, denoting the sharing of 0, 1, or both genes identical by descent. Both methodological approaches reported here used these IBD matrices exclusively for the linkage analyses.

We note that in the process of simulation, recombination could occur only once per chromosome per meiosis and that this recombination could not occur within a gene. This allows us to refer to IBD at a particular gene. In real data, we would not have perfect information, although with extensive genotyping and sequencing we would be able to approach this. Recombination within a gene is also possible, although it is less likely than in the larger intergenic regions. Furthermore, a multipoint analysis is unnecessary because the single point information is completely informative.

Before performing the linkage analyses, we began by making sure that the data had the properties we expected. We believe that this is an important but often neglected step for both real and simulated data. While attempting to identify the population origin of the 202 founders of the 8 pedigrees, we discovered to our surprise that 4,239 SNPs had been altered, although these alterations did not prevent the unambiguous identity and population origin of the founders. We were even more surprised to discover that the founders were not a random sample from the unrelated individuals data set. Instead, all but three of the founders were drawn from the two African samples. Moreover, three of the eight pedigrees were established by approximately equal numbers of founders from the Yoruba and the Luhya population samples. The remaining five pedigrees were primarily founded by one ethnic group or the other. Under ordinary circumstances when using genetic markers for a linkage analysis, it is more cumbersome to analyze families composed of individuals drawn from various ethnic groups because differences in allele frequencies need to be taken into account (see Suarez et al. [1] for an example of combining families of different ancestries in a linkage analysis). Using IBD matrices somewhat obviates this problem, although the possibility looms large that different ancestral populations may be segregating different causative genes.

Accordingly, we decided to concentrate our attention on two groups of kindreds: (A) pedigree 2 (deleting founder 2005 [a Tuscan] and all of her descendants), pedigree 3, and pedigree 4 (deleting founder 4021 [a Yoruba] and all of her descendants); and (B) pedigrees 5 and 6. Individuals in kindred group A are all sampled from the Luhya population, a Bantu ethnic group living primarily in Kenya. It is unclear whether the Luhya sample from the 1000 Genomes Project was randomly sampled from all of the 16 tribes that make up the Luhya ethnic group or from a smaller subset. Individuals in kindred group B are all Yoruba, a large heterogeneous group of about 30 million people from West Africa, predominantly Nigeria.

## Methods

The unusual structure of the data replicates (varying covariates but a constant genetic structure) led us to generate a novel quantitative phenotype from the data as our target of analysis. For a typical variance components linkage analysis, we control for covariates by using the residual after linear regression as a quantitative phenotype. In the linear regression, we model the trait as:(1)

where *α* is the global mean value of the trait, **β** is a vector representing the linear effect sizes of the covariates (**x**), **γ** is a vector representing the linear effect sizes of true (but unknown) causative genetic variants (**g**), and *ε* represents the sum of all other factors. Typically, we analyze:(2)

as the quantitative phenotype, where  and , the maximum-likelihood estimators (MLEs) of the parameters *α* and **β**, are computed from the data and *G* represents the sum of the genetic contributions to the phenotype.

For our analysis, we treated the observations of an individual across multiple data replicates as repeated measures. To do this, we used the framework of a generalized linear mixed model (GLMM) as implemented in PROC GLIMMIX in SAS 9.2 [2] to estimate the MLEs  and  that take the repeated measures into account. As a result, the derived phenotype we used for analysis had a single value for each individual *i* given by:(3)

In this way, each unique genetic observation is counted only once in the analysis, but full information from the repeated measures is used.

For the standard linkage analysis (which does not take into account the repeated measures feature of these data), we used SOLAR [3], and the *p*-values were combined over multiple replicates using Fisher’s method [4]. Smoking, Sex, and Age were used as covariates, although the nonsignificant covariates were not in the final model. We chose not to include Population among the covariates because our analyses were performed separately for the Luhya pedigrees and the Yoruba pedigrees.

## Results

**Figure 1:**
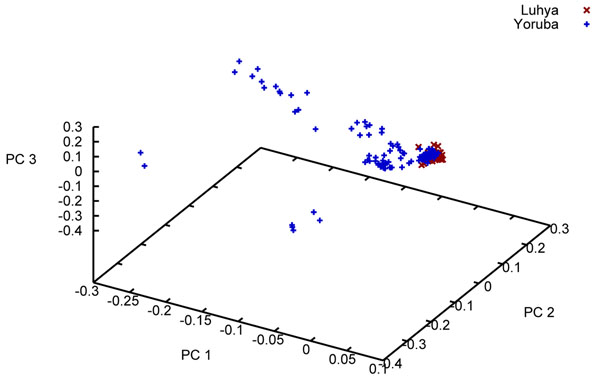
First three principal components from an Eigenstrat analysis of two African populations

We carried out an Eigenstrat analysis [5] using all 24,487 SNPs from the unrelated individuals sample. We observed the expected separation between the broad geographic groups (data not shown). Given the debate surrounding Entine’s [6] controversial book that points out that virtually all the endurance marathon records are held by East Africans while virtually all the shorter speed records are held by West Africans, we were interested to see the degree of clustering between just the Luhya sample and the Yoruba sample (Figure 1). As can be seen, the Luhya sample is tightly clustered for the first three principal components, whereas the Yoruba sample is much more spread out. (By comparison, the two Chinese samples and the Japanese sample are virtually identical [data not shown].)

We applied our two approaches to the first 50 replicates for which SOLAR obtained a convergent solution. The significant results (defined as *p* < 10^−4^ from either method) are reported in Table 1. After receiving the simulation parameters, we divided these into true positives and false positives. For the traditional linkage analysis approach, we list the median *p*-value from the individual replicates, the range of *p*-values, and the *p*-value obtained by combining the data from the families across the 50 replicates (i.e., an analysis based on a sample 50 times the size of a single replicate, *N* = 20,000). The novel repeated measures analysis (performed using the GLMM) is an analysis based on only the individuals from a single replicate (*N* = 400), but it uses the information from 50 repeated measures of each trait to provide a more accurate estimate of the total genetic liability for each individual.

**Table 1:** True positives and false positives from Fisher’s method and GLMM analyses

Trait	Gene	Chromosome	Position	Families	GLMM analysis,	Fisher’s method,		
					−log_10_(p-value)	−log_10_(*p*-value)		
	
						Combined	Min–max	Median
True positives								
Q1	*VEGFA*	6	43,846,955	Luhya	34.30	70.23	0.78–4.95	2.33
Q1	*FLT1*	13	27,775,331	Yoruba	20.85	30.56	0.00–3.47	1.42
Q1	*KDR*	4	55,650,982	Luhya	2.91	5.39	0.00–3.36	0.65
Q1	*VEGFC*	4	177,845,572	Yoruba	3.50	4.43	0.00–3.28	0.58
Q2	*PLAT*	8	42,152,676	Luhya	5.39	4.12	0.00–1.88	0.65
Q2	*SREBF1*	17	17,658,674	Yoruba	4.65	2.44	0.00–2.87	0.45
False positives								
Q1	*LAMB3*	1	207,855,310	Yoruba	1.69	5.07	0.00–2.62	0.67
Q1	*VCAN*	5	82,868,901	Luhya	5.25	15.64	0.00–5.64	1.03
Q1	*UPP1*	7	48,100,910	Luhya	2.12	4.12	0.00–3.53	0.43
Q1	*LMTK2*	7	97,660,051	Yoruba	2.76	4.88	0.00–3.98	0.55
Q1	*CDCA2*	8	24,830,588	Yoruba	5.41	12.10	0.00–3.52	0.73
Q1	*WDR40A*	9	34,097,504	Yoruba	3.40	4.88	0.00–3.40	0.49
Q1	*PPP2R4*	9	130,913,706	Yoruba	2.87	4.08	0.00–2.88	0.47
Q1	*NPTX1*	17	76,060,297	Luhya	8.71	17.20	0.00–3.81	1.11
Q4	*LOC643659*	20	20,004,221	Luhya	0.89	4.29	0.00–3.15	0.49
Q4	*RNF145*	5	159,708,779	Yoruba	4.52	0.65	0.00–2.51	0.00

**Figure 2:**
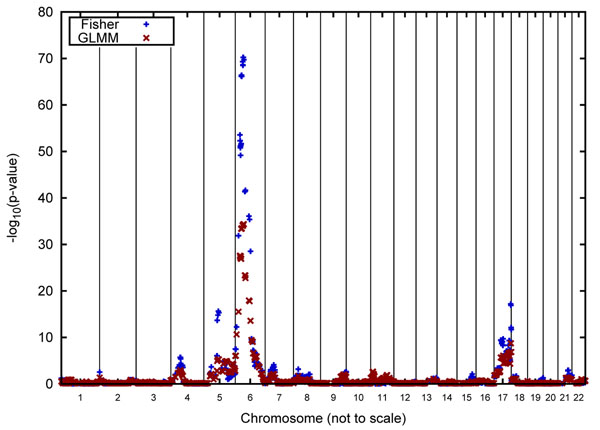
**Linkage analysis for Q1 for families 2, 3, and 4 of the Luhya pedigree**. Genome-wide results of Fisher’s method and GLMM analyses of the Q1 quantitative phenotype in the three Luhya pedigrees. To make the figure more readable, the ratio of the chromosome lengths is not to scale.

**Figure 3:**
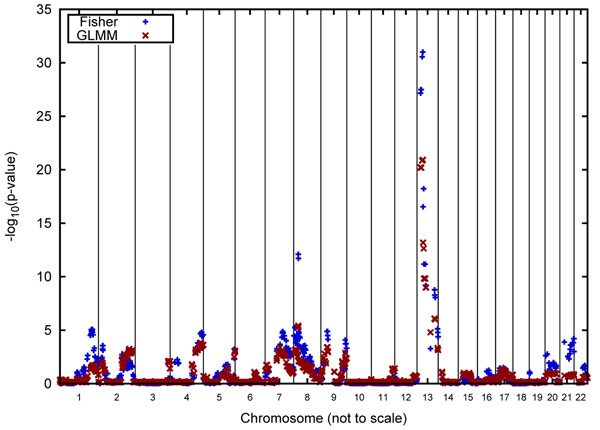
**Linkage analysis for Q1 for families 5 and 6 of the Yoruba pedigree.** Genome-wide results of Fisher’s method and GLMM analyses of the Q1 quantitative phenotype in the two Yoruban pedigrees. To make the figure more readable, the ratio of the chromosome lengths is not to scale.

Figures 2 and 3 graphically report the linkage results for Q1 for the Luhya and Yoruba samples, respectively. Note that these analyses are based on 150 replicate pedigrees for the Luhya sample and 100 replicate pedigrees for the Yoruba sample. Also, because we used the IBD matrices for our single-point analyses, no inference regarding the strength of a linkage signal is made for regions in between the actual genomic positions of the genes.

A striking result of our analyses is the lack of colocalization of linkage signals between the two African groups. Moreover, the maximum signal is often a plateau that occurs over a region of a number of contiguous genes. This appears to be an artifact of the simulation, which allowed only a single recombination per chromosome for each meiosis. Consequently, the position of the recombinations is constant for all of the replicates.

Figures 2 and 3 indicate that the linkage signals obtained with Fisher’s method tend to be higher than those obtained using the GLMM, which, we speculate, is due to the fact that Fisher’s method treats the identical genotypic structure of the 50 replicates as independent observations.

## Discussion and conclusions

The most striking feature revealed by our linkage analysis of the quantitative trait Q1 is that, with one possible exception, none of the five signals seen in the Luhya sample colocalize with any of the four signals found in the Yoruba sample. The exception is the signal seen on chromosome 8 at *CDCA2*, although only the Yoruba signal is highly significant. Had we analyzed each of the three Luhya pedigrees and each of the two Yoruba pedigrees separately, we would have found family-specific signals, all of which would have been stronger than those reported in Table 1 because of our pooling of pedigrees from the two ethnic groups. Clearly, for private polymorphisms or in cases of locus heterogeneity (see, e.g., Morton [7] for the first published example of locus heterogeneity documented by a linkage analysis), linkage analysis of individual families often is preferable to combining all families from a single ethnic group, as we originally did. Indeed, Culverhouse et al. [8], analyzing the unrelated individuals data set, also found that pooling populations could obscure a strong signal even when Population was included as a covariate.

Our Eigenstrat analysis of just the two African samples from the unrelated individuals file (Figure 1) suggests much greater genetic similarity among the Luhya sample than among the Yoruba sample. One possible reason for the apparent heterogeneity of the Yoruba sample is their ancestors’ involvement in the slave trade. Indeed, the Yoruba were one of the largest African groups enslaved and taken by Europeans to the New World, especially Cuba.

It would be naïve to expect any single method to pinpoint all the genetic factors that were simulated, especially for private polymorphisms (i.e., polymorphisms found only in a single family or ethnic group). For instance, Hill et al. [9] recently were able to localize a linkage signal for pleuropulmonary blastoma on the basis of a few small families. However, it took a different method (sequencing) to identify the lesions in the responsible gene (*DICER1*).

The linkage results for Q2 and Q4 (not shown in the figures) did not produce any compelling evidence for the involvement of a well-demarked chromosomal region for these phenotypes. Fisher’s method identified a modest true positive for Q2 on chromosome 8 at *PLAT* and a modest false positive for Q4 on chromosome 20 at *LOC643659*. Similarly, the GLMM identified modest true positives for Q2 at *SREBF1* on chromosome 17 and at *PLAT* and a modest false positive for Q4 on chromosome 5 at *RNF145*.

Other analyses by our group [8] indicate that the four covariates (Sex, Age, Smoking status, and Population) are highly significant for Q4 and explain a large proportion of the variance of Q4. (Over all 200 unrelated replicates, the mean *R*^2^ is 0.787 with a range of 0.759–0.811.) We concluded that Q4 was primarily environmentally determined but that the generating model did include unlinked heritable factors.

Although we correctly located most of the simulated signals for Q1, we also found a number of false-positive signals (Table 1). Two of these (the regions on chromosomes 7 and 9) attained *p*-values of approximately 10^−4^, which normally would not be considered strong evidence of linkage. The other two signals (on chromosomes 8 and 17), however, would be considered persuasive evidence of linkage *if the families were independent*.

**Figure 4:**
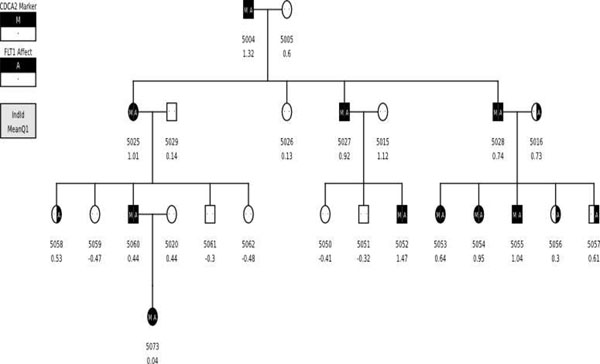
**Subset of pedigree 5** Segment of pedigree 5 showing the cosegregation at C8S775 and C13S434.

We selected the best localized of the two stronger signals and conducted a post hoc analysis of the false-positive signal (i.e., the signal centered over *CDCA2* on chromosome 8) to determine whether there was a reasonable explanation for this strong false-positive finding obtained in the SOLAR analysis. Briefly, we found that this false-positive signal resulted from a chance deviation from random segregation between the A SNP at C8S775 in *CDCA2* (the false-positive signal on chromosome 8) and the C SNP at C13S434 in *FLT1* (a true signal on chromosome 13). Eighteen informative meioses involving these two SNPs were observed in the Yoruba kindred (pedigree 5) responsible for this false-positive signal (Figure 4). Under the hypothesis of linkage between the SNPs at C8S775 and C13S434, these meioses give rise to 15 nonrecombinants (NR) and 3 recombinants (R). Although this deviation from the null expectation of 9NR:9R is marginally significant by a one-sided Fisher’s exact test (*p* = 0.0375), it is not persuasive evidence in a linkage setting. However, when 50 marker-identical families are pooled, this minor nonrandom segregation is telescoped into 750NR:150R, which is a highly significant deviation from the 450NR:450R expected under the hypothesis of no linkage. We believe this is the explanation of the false-positive signal at *CDCA2*.

Finally, we note that the present situation does not reflect any situation observed in human genetics. However, these analyses are appropriate for plant and animal genetics. In particular, homozygous inbred strains and their F1 hybrids offer investigators the ability to observe the characteristics of hundreds of genetically identical organisms in a variety of contexts. Furthermore, the analyses of monozygotic twin data and longitudinal phenotype data are also amenable to analysis with generalized linear models.

## Competing interests

The authors declare that there are no competing interests.

## Authors’ contributions

AH participated in the study design, carried out the analyses, and helped draft the manuscript. RC participated in the study design and helped draft the manuscript. BS participated in the study design and helped draft the manuscript. All authors read and approved the final manuscript.
